# Tetra­iodido[methyl­enebis(diphenyl­phosphine oxide)-κ^2^ *O*:*O*′]tin(IV) chloro­form solvate

**DOI:** 10.1107/S1600536808013627

**Published:** 2008-06-07

**Authors:** Allison M. Caldwell, Joseph M. Tanski

**Affiliations:** a Department of Chemistry, Vassar College, Poughkeepsie, NY 12604, USA

## Abstract

The title compound, [SnI_4_(C_25_H_22_O_2_P_2_)]·CHCl_3_, crystallized from a chloro­form solution of SnI_4_ and the diphosphine CH_2_(PPh_2_)_2_ exposed to air. The monomeric complex displays a distorted octa­hedral coordinaton for the tin(IV) atom with average Sn—I and Sn—O bond lengths of 2.79 (2) and 2.15 (1) Å, respectively.

## Related literature

For examples of structurally characterized tin(IV)–halide complexes of phosphine oxide ligands, see: Genge *et al.* (1999 ▶); Davis *et al.* (2006*a* ▶,*b* ▶); Mohamed *et al.* (2004 ▶). For related literature, see: Levason *et al.* (2003 ▶); Woollins (2003 ▶).
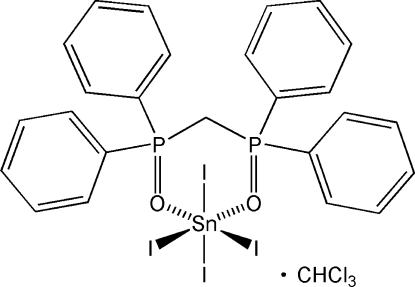


## Experimental

### 

#### Crystal data

[SnI_4_(C_25_H_22_O_2_P_2_)]·CHCl_3_
*M* *_r_* = 1162.02
Monoclinic, 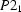

*a* = 9.2639 (5) Å
*b* = 19.0609 (11) Å
*c* = 10.1991 (6) Å
β = 108.479 (1)°
*V* = 1708.08 (17) Å^3^
*Z* = 2
Mo *K*α radiation
μ = 4.71 mm^−1^
*T* = 125 (2) K
0.14 × 0.13 × 0.02 mm

#### Data collection

Bruker APEXII CCD diffractometer
Absorption correction: multi-scan (*SADABS*; Bruker, 2007 ▶) *T*_min_ = 0.558, *T*_max_ = 0.912
20220 measured reflections
7254 independent reflections
6994 reflections with *I* > 2σ(*I*)
*R*_int_ = 0.027

#### Refinement

*R*[*F* ^2^ > 2σ(*F* ^2^)] = 0.021
*wR*(*F* ^2^) = 0.045
*S* = 1.03
7254 reflections
344 parameters
1 restraint
H-atom parameters constrained
Δρ_max_ = 0.73 e Å^−3^
Δρ_min_ = −0.50 e Å^−3^
Absolute structure: Flack (1983 ▶), 3512 Friedel pairs
Flack parameter: 0.004 (14)

### 

Data collection: *APEX2* (Bruker, 2007 ▶); cell refinement: *SAINT* (Bruker, 2007 ▶); data reduction: *SAINT*; program(s) used to solve structure: *SHELXS97* (Sheldrick, 2008 ▶); program(s) used to refine structure: *SHELXL97* (Sheldrick, 2008 ▶); molecular graphics: *SHELXTL* (Sheldrick, 2008 ▶); software used to prepare material for publication: *SHELXTL*.

## Supplementary Material

Crystal structure: contains datablocks global, I. DOI: 10.1107/S1600536808013627/wm2178sup1.cif

Structure factors: contains datablocks I. DOI: 10.1107/S1600536808013627/wm2178Isup2.hkl

Additional supplementary materials:  crystallographic information; 3D view; checkCIF report

## Figures and Tables

**Fig. 1.**
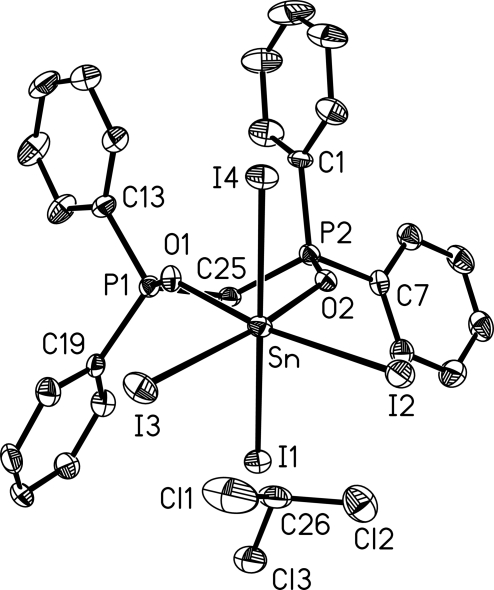
A view of complex (I) with displacement ellipsoids shown at the 50% probability level. H atoms have been omitted for clarity.

## supplementary crystallographic information

### Comment

Tin(IV) iodide may be readily prepared by oxidation of tin metal with iodine (Woollins, 2003). A relatively weak Lewis acid, SnI_4_ nevertheless forms complexes with phosphines and phosphine oxides (Genge *et al.*, 1999; Davis *et al.*,  2006*a*). Similar phosphine and phosphine oxide complexes have been reported of the more Lewis acidic tin(IV) halides, Sn*X*_4_ (*X* = F, Cl, Br; Davis *et al.*,  2006*a*, 2006b; Genge *et al.*, 1999; Mohamed *et al.*,  2004). The phosphine oxide complexes are chiefly obtained by air oxidation of the phosphine ligands in the presence of the tin(IV) halide (Levason *et al.*, 2003).

Reaction of SnI_4_ with CH_2_(PPh_2_)_2_ in CHCl_3_ in the presence of air afforded the title complex [{CH_2_(P(O)Ph_2_)_2_}SnI_4_]^.^CHCl_3_, (I).

Complex (I) exhibits a distorted octahedral coordination at tin. The bis(phosphine oxide) results in a *cis* coordination of the ligand, with Sn—O distances of 2.136 (3) and 2.157 (3) Å, and Sn—I distances of 2.7770 (4), 2.7805 (4), 2.7911 (4) and 2.8199 (4) Å. The smallest bond angle about the pseudooctahedral tin center, 81.1 (1)°, corresponds to the O1—Sn—O2 angle of the chelating bis(phosphine oxide) ligand, with the opposite I2—Sn—I3 angle in the SnO_2_I_2_ plane being the largest, 100.82 (1)°. These distances and angles are similar to those reported for the related bis(phosphine oxide) complex {*o*-C_6_H_4_(P(O)Ph_2_)_2_}SnI_4 _(Genge *et al.*, 1999). The SnO_2_P_2_C six-membered heterocycle in complex (I) is in a distorted boat conformation.

### Experimental

Complex (I) was prepared by treating a chloroform (*ca* 5 ml) solution of SnI_4_ (655 mg, 1.1 mmol) with an excess of CH_2_(PPh_2_)_2_ (922 mg, 2.6 mmol) in the presence of air. Suitable crystals for single crystal X-ray analysis formed within 1 week at room temperature. A small sample of crystals was separated for structural analysis, and the remaining crystals were collected by filtration on a glass frit, washed three times with *ca* 5 ml of chloroform, and dried briefly under vacuum (yield 659 mg (54%) of a red-orange product). Elemental analysis confirmed that the compound was obtained as the chloroform solvate: anal. calcd. for (I) C, 26.87%; H, 1.99%; N, 0.00%. Found C, 26.97%; H, 1.73%; N, <0.02%. (Elemental analysis performed by Robertson Microlit Laboratories, Madison, NJ, USA.)

### Refinement

H atoms on carbon were included in calculated positions and were refined using a riding model with U_iso_(H) = 1.2U_eq_(C) of the parent atom.

### Crystal data

**Table:**

[SnI_4_(C_25_H_22_O_2_P_2_)]·CHCl_3_	*F*_000_ = 1076
*M**_r_* = 1162.02	*D*_x_ = 2.259 Mg m^−^^3^
Monoclinic, *P*2_1_	Mo *K*α radiation λ = 0.71073 Å
Hall symbol: P 2yb	Cell parameters from 9838 reflections
*a* = 9.2639 (5) Å	θ = 2.3–28.3º
*b* = 19.0609 (11) Å	µ = 4.71 mm^−^^1^
*c* = 10.1991 (6) Å	*T* = 125 (2) K
β = 108.479 (1)º	Plate, orange
*V* = 1708.08 (17) Å^3^	0.14 × 0.13 × 0.02 mm
*Z* = 2	

### Data collection

**Table:**

Bruker APEXII CCD diffractometer	7254 independent reflections
Radiation source: fine-focus sealed tube	6994 reflections with *I* > 2σ(*I*)
Monochromator: graphite	*R*_int_ = 0.027
*T* = 125(2) K	θ_max_ = 26.7º
φ and ω scans	θ_min_ = 2.1º
Absorption correction: multi-scan(SADABS; Bruker, 2007)	*h* = −11→11
*T*_min_ = 0.558, *T*_max_ = 0.912	*k* = −24→24
20220 measured reflections	*l* = −12→12

### Refinement

**Table:**

Refinement on *F*^2^	Hydrogen site location: inferred from neighbouring sites
Least-squares matrix: full	H-atom parameters constrained
*R*[*F*^2^ > 2σ(*F*^2^)] = 0.021	*w* = 1/[σ^2^(*F*_o_^2^) + (0.0195*P*)^2^ + 0.2278*P*] where *P* = (*F*_o_^2^ + 2*F*_c_^2^)/3
*wR*(*F*^2^) = 0.046	(Δ/σ)_max_ = 0.002
*S* = 1.03	Δρ_max_ = 0.73 e Å^−^^3^
7254 reflections	Δρ_min_ = −0.50 e Å^−^^3^
344 parameters	Extinction correction: none
1 restraint	Absolute structure: Flack (1983), 3512 Friedel pairs
Primary atom site location: structure-invariant direct methods	Flack parameter: 0.004 (14)
Secondary atom site location: difference Fourier map	

### Special details

**Table:**

Geometry. All e.s.d.'s (except the e.s.d. in the dihedral angle between two l.s. planes) are estimated using the full covariance matrix. The cell e.s.d.'s are taken into account individually in the estimation of e.s.d.'s in distances, angles and torsion angles; correlations between e.s.d.'s in cell parameters are only used when they are defined by crystal symmetry. An approximate (isotropic) treatment of cell e.s.d.'s is used for estimating e.s.d.'s involving l.s. planes.
Refinement. A suitable crystal was mounted in a nylon loop with Paratone-*N* cryoprotectant oil and data was collected on a Bruker *APEX* 2 CCD platform diffractometer. The data was cut off at 0.79 Å because data in the highest resolution range (0.75-0.78 Å) was very incomplete, acting to reduce the overall completeness to 99.4%. The structure was solved using direct methods and standard difference map techniques, and was refined by full-matrix least-squares procedures on F^2^ with *SHELXTL* Version 6.14 (Sheldrick, 2008). One least squares restraint is required for the floating origin of space group *P*2_1_. All non-hydrogen atoms were refined anisotropically. Refinement of *F*^2^ against ALL reflections. The weighted *R*-factor *wR* and goodness of fit *S* are based on *F*^2^, conventional *R*-factors *R* are based on *F*, with *F* set to zero for negative *F*^2^. The threshold expression of *F*^2^ > σ(*F*^2^) is used only for calculating *R*-factors(gt) *etc*. and is not relevant to the choice of reflections for refinement. *R*-factors based on *F*^2^ are statistically about twice as large as those based on *F*, and *R*-factors based on ALL data will be even larger. EXTI refined to zero and was removed from the refinement.

### Fractional atomic coordinates and isotropic or equivalent isotropic displacement parameters (Å^2^)

**Table:**

	*x*	*y*	*z*	*U*_iso_*/*U*_eq_	
Sn	0.83582 (3)	0.791335 (15)	0.25375 (3)	0.01631 (6)	
I1	0.75473 (3)	0.673729 (13)	0.38291 (3)	0.02340 (7)	
I2	0.98608 (3)	0.706487 (16)	0.11538 (3)	0.02731 (7)	
I3	1.07128 (3)	0.821230 (17)	0.49436 (3)	0.02768 (7)	
I4	0.89066 (3)	0.910079 (14)	0.11840 (3)	0.02389 (7)	
P1	0.54635 (12)	0.85261 (5)	0.35831 (11)	0.0155 (2)	
P2	0.45533 (11)	0.79067 (6)	0.06921 (10)	0.0162 (2)	
O1	0.6960 (3)	0.85997 (14)	0.3271 (3)	0.0178 (6)	
O2	0.6235 (3)	0.77647 (14)	0.0902 (3)	0.0166 (6)	
C1	0.3884 (5)	0.8709 (2)	−0.0204 (4)	0.0193 (9)	
C2	0.2576 (5)	0.9026 (3)	−0.0116 (5)	0.0329 (11)	
H2A	0.2042	0.8827	0.0450	0.039*	
C3	0.2044 (6)	0.9636 (3)	−0.0854 (6)	0.0420 (13)	
H3A	0.1151	0.9859	−0.0789	0.050*	
C4	0.2817 (7)	0.9918 (3)	−0.1683 (6)	0.0487 (15)	
H4A	0.2453	1.0336	−0.2187	0.058*	
C5	0.4107 (6)	0.9603 (3)	−0.1788 (5)	0.0372 (13)	
H5A	0.4625	0.9798	−0.2371	0.045*	
C6	0.4650 (5)	0.9000 (3)	−0.1042 (4)	0.0272 (10)	
H6A	0.5551	0.8784	−0.1102	0.033*	
C7	0.3424 (5)	0.7198 (2)	−0.0266 (4)	0.0187 (9)	
C8	0.2165 (5)	0.7324 (3)	−0.1412 (4)	0.0269 (10)	
H8A	0.1873	0.7791	−0.1699	0.032*	
C9	0.1332 (5)	0.6761 (3)	−0.2139 (4)	0.0316 (11)	
H9A	0.0471	0.6843	−0.2929	0.038*	
C10	0.1755 (6)	0.6084 (3)	−0.1715 (5)	0.0303 (11)	
H10A	0.1178	0.5701	−0.2210	0.036*	
C11	0.3025 (6)	0.5957 (3)	−0.0562 (5)	0.0322 (11)	
H11A	0.3310	0.5490	−0.0267	0.039*	
C12	0.3863 (5)	0.6516 (2)	0.0146 (5)	0.0253 (10)	
H12A	0.4744	0.6433	0.0918	0.030*	
C13	0.4575 (5)	0.9369 (2)	0.3409 (4)	0.0194 (9)	
C14	0.3480 (5)	0.9506 (2)	0.4051 (5)	0.0280 (10)	
H14A	0.3271	0.9166	0.4649	0.034*	
C15	0.2702 (5)	1.0132 (3)	0.3818 (6)	0.0341 (12)	
H15A	0.1950	1.0220	0.4249	0.041*	
C16	0.3002 (6)	1.0628 (2)	0.2971 (5)	0.0345 (12)	
H16A	0.2462	1.1059	0.2820	0.041*	
C17	0.4098 (6)	1.0500 (2)	0.2331 (5)	0.0320 (11)	
H17A	0.4297	1.0841	0.1732	0.038*	
C18	0.4897 (5)	0.9874 (2)	0.2569 (5)	0.0255 (10)	
H18A	0.5667	0.9792	0.2156	0.031*	
C19	0.5743 (5)	0.8157 (2)	0.5259 (4)	0.0181 (8)	
C20	0.4710 (5)	0.7703 (2)	0.5528 (4)	0.0218 (9)	
H20A	0.3760	0.7615	0.4841	0.026*	
C21	0.5063 (5)	0.7376 (2)	0.6807 (4)	0.0244 (10)	
H21A	0.4348	0.7069	0.6998	0.029*	
C22	0.6457 (6)	0.7497 (2)	0.7807 (4)	0.0285 (10)	
H22A	0.6704	0.7264	0.8674	0.034*	
C23	0.7478 (5)	0.7954 (2)	0.7542 (4)	0.0272 (10)	
H23A	0.8426	0.8038	0.8235	0.033*	
C24	0.7148 (5)	0.8295 (2)	0.6282 (4)	0.0255 (9)	
H24A	0.7853	0.8615	0.6110	0.031*	
C25	0.4202 (4)	0.7925 (2)	0.2343 (4)	0.0168 (8)	
H25A	0.4335	0.7447	0.2740	0.020*	
H25B	0.3133	0.8067	0.2192	0.020*	
C26	0.1709 (7)	0.5583 (3)	0.3620 (5)	0.0447 (14)	
H26A	0.0865	0.5924	0.3219	0.054*	
Cl1	0.3366 (3)	0.60552 (10)	0.4432 (2)	0.1039 (9)	
Cl2	0.19826 (17)	0.50905 (9)	0.22600 (15)	0.0518 (4)	
Cl3	0.11844 (19)	0.50376 (8)	0.47642 (15)	0.0522 (4)	

### Atomic displacement parameters (Å^2^)

**Table:**

	*U*^11^	*U*^22^	*U*^33^	*U*^12^	*U*^13^	*U*^23^
Sn	0.01542 (13)	0.01735 (13)	0.01582 (13)	0.00235 (11)	0.00450 (10)	0.00282 (10)
I1	0.02927 (16)	0.01839 (14)	0.02235 (14)	0.00030 (12)	0.00790 (12)	0.00269 (11)
I2	0.03002 (16)	0.03117 (15)	0.02356 (14)	0.01382 (13)	0.01252 (12)	0.00496 (13)
I3	0.01861 (14)	0.03691 (17)	0.02288 (14)	−0.00201 (13)	0.00001 (11)	0.00444 (13)
I4	0.02432 (14)	0.02220 (14)	0.02621 (15)	−0.00028 (12)	0.00951 (12)	0.00677 (11)
P1	0.0163 (5)	0.0144 (5)	0.0159 (5)	−0.0010 (4)	0.0053 (4)	−0.0008 (4)
P2	0.0160 (5)	0.0178 (5)	0.0132 (5)	0.0002 (5)	0.0027 (4)	0.0005 (4)
O1	0.0178 (14)	0.0193 (15)	0.0154 (14)	−0.0023 (11)	0.0041 (11)	−0.0042 (11)
O2	0.0173 (14)	0.0179 (15)	0.0133 (13)	0.0013 (11)	0.0029 (11)	0.0007 (11)
C1	0.024 (2)	0.015 (2)	0.016 (2)	−0.0008 (17)	0.0017 (17)	0.0056 (16)
C2	0.021 (2)	0.037 (3)	0.038 (3)	0.006 (2)	0.006 (2)	0.009 (2)
C3	0.035 (3)	0.037 (3)	0.050 (3)	0.012 (2)	0.006 (3)	0.019 (3)
C4	0.037 (3)	0.035 (3)	0.060 (4)	0.009 (2)	−0.005 (3)	0.023 (3)
C5	0.034 (3)	0.036 (3)	0.035 (3)	−0.009 (2)	0.003 (2)	0.017 (2)
C6	0.020 (2)	0.034 (3)	0.024 (2)	−0.001 (2)	0.0028 (18)	0.007 (2)
C7	0.018 (2)	0.022 (2)	0.0162 (19)	−0.0016 (17)	0.0069 (16)	−0.0033 (17)
C8	0.029 (3)	0.027 (2)	0.021 (2)	−0.006 (2)	0.0034 (19)	−0.0019 (18)
C9	0.030 (3)	0.040 (3)	0.018 (2)	−0.006 (2)	−0.0014 (19)	0.000 (2)
C10	0.033 (3)	0.033 (3)	0.023 (2)	−0.011 (2)	0.005 (2)	−0.007 (2)
C11	0.038 (3)	0.025 (3)	0.030 (3)	−0.003 (2)	0.006 (2)	−0.002 (2)
C12	0.029 (3)	0.023 (2)	0.019 (2)	−0.0035 (19)	0.0013 (19)	−0.0034 (18)
C13	0.017 (2)	0.0147 (19)	0.023 (2)	0.0010 (16)	0.0021 (17)	−0.0006 (17)
C14	0.026 (2)	0.020 (2)	0.043 (3)	−0.0018 (19)	0.019 (2)	−0.005 (2)
C15	0.026 (3)	0.026 (3)	0.058 (3)	0.000 (2)	0.025 (2)	−0.008 (2)
C16	0.035 (3)	0.016 (2)	0.046 (3)	0.006 (2)	0.004 (2)	−0.002 (2)
C17	0.040 (3)	0.023 (2)	0.033 (3)	0.000 (2)	0.012 (2)	0.004 (2)
C18	0.030 (2)	0.020 (2)	0.026 (2)	−0.0014 (19)	0.010 (2)	−0.0008 (18)
C19	0.025 (2)	0.016 (2)	0.0158 (19)	0.0014 (17)	0.0098 (17)	−0.0028 (16)
C20	0.017 (2)	0.025 (2)	0.024 (2)	−0.0006 (17)	0.0089 (18)	−0.0023 (17)
C21	0.028 (2)	0.027 (2)	0.023 (2)	0.0009 (19)	0.0145 (19)	0.0016 (18)
C22	0.046 (3)	0.027 (2)	0.014 (2)	0.005 (2)	0.011 (2)	0.0030 (18)
C23	0.032 (3)	0.033 (3)	0.015 (2)	0.004 (2)	0.0050 (18)	−0.0048 (19)
C24	0.026 (2)	0.026 (2)	0.022 (2)	−0.0040 (19)	0.0036 (18)	−0.0037 (19)
C25	0.0190 (19)	0.0148 (18)	0.0162 (19)	−0.0010 (18)	0.0048 (15)	0.0025 (17)
C26	0.059 (4)	0.027 (3)	0.036 (3)	−0.003 (3)	−0.002 (3)	0.010 (2)
Cl1	0.1176 (17)	0.0573 (12)	0.0806 (14)	−0.0501 (12)	−0.0484 (12)	0.0197 (10)
Cl2	0.0441 (8)	0.0688 (10)	0.0441 (8)	0.0007 (7)	0.0161 (7)	0.0017 (7)
Cl3	0.0719 (10)	0.0461 (8)	0.0415 (8)	0.0182 (8)	0.0221 (7)	0.0119 (7)

### Geometric parameters (Å, °)

**Table:**

Sn—O1	2.136 (3)	C10—H10A	0.9500
Sn—O2	2.157 (3)	C11—C12	1.379 (6)
Sn—I3	2.7770 (4)	C11—H11A	0.9500
Sn—I4	2.7805 (4)	C12—H12A	0.9500
Sn—I2	2.7911 (4)	C13—C18	1.383 (6)
Sn—I1	2.8199 (4)	C13—C14	1.395 (6)
P1—O1	1.524 (3)	C14—C15	1.375 (7)
P1—C13	1.788 (4)	C14—H14A	0.9500
P1—C19	1.790 (4)	C15—C16	1.366 (7)
P1—C25	1.828 (4)	C15—H15A	0.9500
P2—O2	1.527 (3)	C16—C17	1.392 (7)
P2—C1	1.788 (4)	C16—H16A	0.9500
P2—C7	1.795 (4)	C17—C18	1.383 (6)
P2—C25	1.814 (4)	C17—H17A	0.9500
C1—C2	1.383 (6)	C18—H18A	0.9500
C1—C6	1.388 (6)	C19—C20	1.381 (6)
C2—C3	1.389 (7)	C19—C24	1.411 (6)
C2—H2A	0.9500	C20—C21	1.387 (6)
C3—C4	1.378 (8)	C20—H20A	0.9500
C3—H3A	0.9500	C21—C22	1.388 (6)
C4—C5	1.372 (8)	C21—H21A	0.9500
C4—H4A	0.9500	C22—C23	1.375 (7)
C5—C6	1.382 (7)	C22—H22A	0.9500
C5—H5A	0.9500	C23—C24	1.385 (6)
C6—H6A	0.9500	C23—H23A	0.9500
C7—C8	1.386 (6)	C24—H24A	0.9500
C7—C12	1.387 (6)	C25—H25A	0.9900
C8—C9	1.391 (6)	C25—H25B	0.9900
C8—H8A	0.9500	C26—Cl3	1.742 (6)
C9—C10	1.377 (7)	C26—Cl1	1.746 (6)
C9—H9A	0.9500	C26—Cl2	1.758 (6)
C10—C11	1.397 (7)	C26—H26A	1.0000
			
O1—Sn—O2	81.11 (10)	C9—C10—H10A	119.8
O1—Sn—I3	87.62 (7)	C11—C10—H10A	119.8
O2—Sn—I3	168.16 (7)	C12—C11—C10	119.4 (4)
O1—Sn—I4	84.36 (8)	C12—C11—H11A	120.3
O2—Sn—I4	89.34 (7)	C10—C11—H11A	120.3
I3—Sn—I4	93.196 (12)	C11—C12—C7	120.2 (4)
O1—Sn—I2	170.71 (7)	C11—C12—H12A	119.9
O2—Sn—I2	90.68 (7)	C7—C12—H12A	119.9
I3—Sn—I2	100.824 (13)	C18—C13—C14	119.5 (4)
I4—Sn—I2	91.247 (12)	C18—C13—P1	120.3 (3)
O1—Sn—I1	92.43 (8)	C14—C13—P1	120.1 (3)
O2—Sn—I1	86.79 (7)	C15—C14—C13	120.0 (4)
I3—Sn—I1	90.070 (12)	C15—C14—H14A	120.0
I4—Sn—I1	175.320 (14)	C13—C14—H14A	120.0
I2—Sn—I1	91.431 (12)	C16—C15—C14	120.6 (4)
O1—P1—C13	108.51 (18)	C16—C15—H15A	119.7
O1—P1—C19	111.71 (18)	C14—C15—H15A	119.7
C13—P1—C19	111.74 (19)	C15—C16—C17	120.0 (4)
O1—P1—C25	109.74 (17)	C15—C16—H16A	120.0
C13—P1—C25	108.71 (19)	C17—C16—H16A	120.0
C19—P1—C25	106.37 (19)	C18—C17—C16	119.9 (4)
O2—P2—C1	113.34 (18)	C18—C17—H17A	120.1
O2—P2—C7	109.60 (18)	C16—C17—H17A	120.1
C1—P2—C7	108.48 (19)	C13—C18—C17	120.0 (4)
O2—P2—C25	110.39 (17)	C13—C18—H18A	120.0
C1—P2—C25	108.8 (2)	C17—C18—H18A	120.0
C7—P2—C25	105.94 (19)	C20—C19—C24	120.3 (4)
P1—O1—Sn	135.20 (17)	C20—C19—P1	122.4 (3)
P2—O2—Sn	136.45 (16)	C24—C19—P1	117.0 (3)
C2—C1—C6	119.7 (4)	C19—C20—C21	119.7 (4)
C2—C1—P2	120.7 (3)	C19—C20—H20A	120.1
C6—C1—P2	119.6 (3)	C21—C20—H20A	120.1
C1—C2—C3	119.9 (5)	C20—C21—C22	120.3 (4)
C1—C2—H2A	120.0	C20—C21—H21A	119.8
C3—C2—H2A	120.0	C22—C21—H21A	119.8
C4—C3—C2	119.7 (5)	C23—C22—C21	119.9 (4)
C4—C3—H3A	120.2	C23—C22—H22A	120.1
C2—C3—H3A	120.2	C21—C22—H22A	120.1
C5—C4—C3	120.8 (5)	C22—C23—C24	121.1 (4)
C5—C4—H4A	119.6	C22—C23—H23A	119.5
C3—C4—H4A	119.6	C24—C23—H23A	119.5
C4—C5—C6	119.7 (5)	C23—C24—C19	118.7 (4)
C4—C5—H5A	120.1	C23—C24—H24A	120.7
C6—C5—H5A	120.1	C19—C24—H24A	120.7
C5—C6—C1	120.2 (4)	P2—C25—P1	113.0 (2)
C5—C6—H6A	119.9	P2—C25—H25A	109.0
C1—C6—H6A	119.9	P1—C25—H25A	109.0
C8—C7—C12	120.3 (4)	P2—C25—H25B	109.0
C8—C7—P2	121.2 (3)	P1—C25—H25B	109.0
C12—C7—P2	118.4 (3)	H25A—C25—H25B	107.8
C7—C8—C9	119.5 (4)	Cl3—C26—Cl1	112.4 (3)
C7—C8—H8A	120.2	Cl3—C26—Cl2	110.5 (3)
C9—C8—H8A	120.2	Cl1—C26—Cl2	108.9 (4)
C10—C9—C8	120.0 (4)	Cl3—C26—H26A	108.3
C10—C9—H9A	120.0	Cl1—C26—H26A	108.3
C8—C9—H9A	120.0	Cl2—C26—H26A	108.3
C9—C10—C11	120.4 (4)		
			
C13—P1—O1—Sn	−154.2 (2)	C9—C10—C11—C12	−0.5 (8)
C19—P1—O1—Sn	82.2 (3)	C10—C11—C12—C7	1.6 (7)
C25—P1—O1—Sn	−35.5 (3)	C8—C7—C12—C11	−1.7 (7)
O2—Sn—O1—P1	54.1 (2)	P2—C7—C12—C11	179.9 (4)
I3—Sn—O1—P1	−122.2 (2)	O1—P1—C13—C18	24.9 (4)
I4—Sn—O1—P1	144.3 (2)	C19—P1—C13—C18	148.5 (4)
I1—Sn—O1—P1	−32.3 (2)	C25—P1—C13—C18	−94.4 (4)
C1—P2—O2—Sn	97.2 (3)	O1—P1—C13—C14	−159.1 (3)
C7—P2—O2—Sn	−141.4 (2)	C19—P1—C13—C14	−35.5 (4)
C25—P2—O2—Sn	−25.1 (3)	C25—P1—C13—C14	81.6 (4)
O1—Sn—O2—P2	−16.7 (2)	C18—C13—C14—C15	1.7 (7)
I3—Sn—O2—P2	1.4 (6)	P1—C13—C14—C15	−174.3 (4)
I4—Sn—O2—P2	−101.1 (2)	C13—C14—C15—C16	−0.7 (8)
I2—Sn—O2—P2	167.6 (2)	C14—C15—C16—C17	0.3 (8)
I1—Sn—O2—P2	76.2 (2)	C15—C16—C17—C18	−0.9 (8)
O2—P2—C1—C2	−159.9 (3)	C14—C13—C18—C17	−2.3 (7)
C7—P2—C1—C2	78.1 (4)	P1—C13—C18—C17	173.7 (3)
C25—P2—C1—C2	−36.7 (4)	C16—C17—C18—C13	1.9 (7)
O2—P2—C1—C6	22.7 (4)	O1—P1—C19—C20	−144.0 (3)
C7—P2—C1—C6	−99.3 (4)	C13—P1—C19—C20	94.2 (4)
C25—P2—C1—C6	145.9 (3)	C25—P1—C19—C20	−24.3 (4)
C6—C1—C2—C3	−0.5 (7)	O1—P1—C19—C24	30.0 (4)
P2—C1—C2—C3	−177.9 (4)	C13—P1—C19—C24	−91.8 (4)
C1—C2—C3—C4	0.6 (8)	C25—P1—C19—C24	149.7 (3)
C2—C3—C4—C5	0.1 (9)	C24—C19—C20—C21	−0.6 (6)
C3—C4—C5—C6	−0.8 (9)	P1—C19—C20—C21	173.2 (3)
C4—C5—C6—C1	0.9 (8)	C19—C20—C21—C22	−0.8 (7)
C2—C1—C6—C5	−0.3 (7)	C20—C21—C22—C23	1.5 (7)
P2—C1—C6—C5	177.1 (4)	C21—C22—C23—C24	−0.6 (7)
O2—P2—C7—C8	−130.5 (4)	C22—C23—C24—C19	−0.8 (7)
C1—P2—C7—C8	−6.3 (4)	C20—C19—C24—C23	1.4 (6)
C25—P2—C7—C8	110.4 (4)	P1—C19—C24—C23	−172.7 (3)
O2—P2—C7—C12	47.8 (4)	O2—P2—C25—P1	52.7 (3)
C1—P2—C7—C12	172.0 (3)	C1—P2—C25—P1	−72.2 (3)
C25—P2—C7—C12	−71.3 (4)	C7—P2—C25—P1	171.3 (2)
C12—C7—C8—C9	0.8 (7)	O1—P1—C25—P2	−27.7 (3)
P2—C7—C8—C9	179.1 (3)	C13—P1—C25—P2	90.8 (3)
C7—C8—C9—C10	0.3 (7)	C19—P1—C25—P2	−148.7 (2)
C8—C9—C10—C11	−0.5 (7)		
